# Profound alterations of cancer transcriptomes by the RNase L inhibitor ABCE1 through the modulation of UU/UA-dinucleotide rich transcript abundance

**DOI:** 10.1080/15476286.2026.2629475

**Published:** 2026-02-09

**Authors:** Edward Hitti, Tala Bakheet, Linah Mahmoud, Nada Al-Mutairi, Latifa Alhaj, Fahad Al-Zoghaibi, Khalid S. A. Khabar

**Affiliations:** Molecular BioMedicine Department, Research and Innovation, King Faisal Specialist Hospital and Research Centre, Riyadh, Saudi Arabia

**Keywords:** ABCE1, RNase L, colorectal adenocarcinoma, lung adenocarcinoma, UU/UA dinucleotides

## Abstract

Tumorigenesis is commonly driven by genetic mutations and disruptions in cellular signalling pathways. Here we show that the oncogenic overexpression of the RNase L inhibitor ABCE1, a component of interferon signalling, leads to distinct and extensive deviations in cancer transcriptomes. RNase L is a cellular endonuclease that cleaves RNA molecules at specific UU and UA dinucleotide sites. Typically, it is activated by viral infections and interferon signalling leading to targeting and destruction of UU/UA-rich viral and cellular mRNA. RNase L has also homoeostatic and tumour suppressive roles. Relying on patient transcriptomic data, we show that ABCE1 is extensively overexpressed in colorectal cancer (CRC) and to a lesser extent in lung cancer. This upregulation was strongly associated with the co-upregulation of almost all UU/UA rich transcripts and downregulation of those that are UU/UA-poor. Many of upregulated mRNAs code for proteins involved in cell cycle regulation and mitosis. Accordingly, the knockdown of ABCE1 in the CRC cell line HT29 led to reduced proliferation. Surprisingly, the very high ABCE1 levels were associated with improved patient survival in CRC. This observation might be related to an anti-ABCE1-specific immune response due to the induction of tumour-reactive cytotoxic T lymphocytes by ABCE1 as previously reported. In lung cancer ABCE1 overexpression is milder and is associated with poor survival. We report a measurable, specific, and extensive modulation of cancer transcriptomes by the oncogenic overexpression of a component of interferon signalling with unexpected outcomes on patient survival.

## Introduction

The human endonuclease RNase L is a cellular enzyme that cleaves single stranded RNA with preference to UU and UA sites (UU+UA) [1]. It has a well-established role in anti-viral innate immunity; viral infections activate RNase L by a multistep mechanism that includes the induction of oligo-adenylate synthetases (OASs) which synthesize 2′-5′ linked oligo-adenylates (2-5A). 2-5As bind to and activate RNase L; the active nuclease defends the host by cleaving viral and cellular RNAs at UU+UA sites leading to the inhibition of viral replication [2–4]. RNase L activity is not limited to innate immune responses; a homoeostatic role has also been proposed, that includes anti-proliferative, apoptotic and other tumour suppressive activities [5]. Mutations that reduce its ability to degrade RNA predispose men to an increased incidence of prostate cancer [6]. A prostate-cancer-susceptibility variant of RNase L, namely, with the Arg462Gln was also associated with age of onset of hereditary non-polyposis colorectal cancer (CRC) in a dose-dependent way [7]. Selective cellular RNA targets of RNase L include the double-stranded protein kinase, PKR, and the RNA binding protein HuR [8,9]. Rath et al. identified hundreds of putative RNase L targets including suppressors of mammalian cell adhesion and proliferation [10]. Reducing UU+UA sites in the coding region of transcripts is sufficient for increasing their stability and the expression of recombinant genes [11].

RLI or ABCE1 is a cellular inhibitor of RNase L, and is subsequently involved in the same processes of innate immunity and tissue homoeostasis [12,13]. In contrast to RNase L, ABCE1 is often upregulated in tumours and its expression has been linked to cancer progression and metastasis in lung cancer [14–16]. The Konckdown of ABCE1 by siRNA leads to a reduction of the proliferation in small cell lung cancer and the accumulation of human cells in the S-phase. ABCE1 also has RNase L-independent post-transcriptional roles, including ribosome biogenesis and the initiation of eukaryotic mRNA translation [17].

Here, we performed a search for UU+UA dinucleotides in the human transcriptome and determined the UU+UA ratios of available protein coding human transcripts. Utilizing the Cancer Genome Atlas Program (TCGA) PanCancer atlas data, we were able to correlate the abundance of cancer transcripts with RNase L and ABCE1 mRNAs levels. The results indicate that the RNase L/ABCE1 axis has a profound effect on cancer transcriptomes; modulating the expression of hundreds of genes based on their UU+UA ratios. We observed a true ‘transcriptomic shift’ that is likely the result of the oncogenic upregulation of a single factor, namely ABCE1. Amongst the upregulated genes is a set of UU+U rich transcripts that are involved in mitosis and cell cycle control. We show that oncogenic ABCE1 overexpression enhances the proliferation in CRC cancer cells but has unexpected effects on survival of CRC patients.

## Methods

### Generation of UU+UA ratios in human protein coding genes

cDNA, 5′UTR, 3′UTR and CDS sequences of the human genome were downloaded from Ensembl BioMart (https://useast.ensembl.org/biomart/martview/) (RRID:SCR_002344). Ensembl genes 113 database and the dataset of human genes (GRCh38.p13) were selected. We selected the longest transcript for each gene from a total of 19,308 protein-coding genes. In the attributes section, the sequences option was selected, and the regions filter of 3′UTR, 5′UTR, CDS or cDNA was separately saved as a FASTA file respectively. Compseq program from the package of the European Molecular Biology open software suite (EMBOSS, RRID: SCR_008493) was used to determine the frequency of UU and UA with the formula: Observed frequency = observed counts/total counts. To run Compseq, each gene needs to be in a separate file. Therefore, the FASTA file was split into separate multiple FASTA file formats for each gene area. For the cDNA region, multiple transcripts were assigned to single gene. Comprehensive criteria were set to choose the longest transcript for each mRNA using PERL code. Compseq was used to detect all possible dinucleotides (AA, AC, AG, AU, CC, GG, CG, CU, GA, GG, GU, UC, UG, UU, UA). Linux commands were written to extract these ratios from each file and the frequency of UU and UA were added to get the total numbers of UU+UA for each gene. This resulted in lists of UU+UA ratios of mRNAs (Supplemental Table 1).

### Bioinformatics analysis, transcriptomic and statistics tools

The cBioportal website (cbioportal.org, RRID:SCR_014555) was used for the analysis of RNA-seq gene expression levels and correlations with ABCE1 [18,19]. We investigated cancer types that have TCGA PanCancer atlas data, including breast invasive carcinoma (Breast), colorectal adenocarcinoma (Colorectal or CRC), liver hepatocellular carcinoma (Liver), lung adenocarcinoma (Lung), prostate adenocarcinoma (Prostate) and thyroid carcinoma (Thyroid). The levels of RNase L and ABCE1 mRNAs relative to normal samples were determined by selecting the function “mRNA expression z-scores relative to normal samples (log RNA Seq V2 RSEM), the levels were downloaded from every tested cancer and plotted on dot plots in GraphPad prism. On the other hand, ‘mRNA expression z-scores relative to all samples’ was selected to compare the UU+UA ratios of the mRNAs that co-upregulate or co-downregulate with RNase L or ABCE1. We set a significant (Q < 0.05) overexpression threshold of 1.5 standard deviations (SD) above the mean for RNase L or ABCE1 overexpression and −1.5 standard deviations (SD) below the mean for RNase L or ABCE1 under-expression. The UU+UA ratios for the selected were plotted on dot plots in graphpad prism.

The Pearson correlations of all mRNAs with ABCE1 mRNA in the tested cancers, along with its significance (p-adjusted), were obtained from cBioportal. Kaplan – Meier survival curves were plotted in the ‘kmplot.com’ portal by selecting the following criteria: best cut-off and JetSet probe, further specific selections are stated in the text and figure legends [20]. The Shiny Go 0.82 portal (http://bioinformatics.sdstate.edu/go/) was used to generate gene enrichments and display based on interactome analysis. Graphpad prism was used to draw volcano plots, perform statistical analysis and display as indicated in the figure legends [21,22].

### Cell lines, ABCE1 expression vectors, ABCE1 KD, and real time PCR and western blotting

HEK293, HCT116, Caco-2 and HT29 cells were ordered from ATCC and grown in DMEM medium supplemented with 10% FBS and 1% antibiotics and 1% L-Glutamine (Sigma-Aldrich). CCD841CON was grown in DMEM F12 supplemented with EGF (5 ng/ml), hydrocortisone (0.5 ng/ml), and insulin (10ug/ml) and 15% FBS. HA-tagged ABCE1 expression vector was ordered from Genecopoeia DNA, pcDNA3.1 empty vector was ordered from Thermofisher. HEK293 cells were transfected with 3 µg DNA in six well plates using Lipofectamine LTX (Thermofisher). Konckdown with siRNA was performed as previously described [23]. Briefly, siRNAs were custom synthesized by Metabion (Martinsried, Bavaria, Germany). The control SCR RNA is Sense: 5GAUAUGUCAACUCAGUACUtt and antisense: 5′-AGUACUGAGUUG ACAUAUCtt siRNA. For ABCE1 two siRNAs were used: first siRNA sense GAU GGU GCC UAA GCA UCU Att, Antisense: UAG CUG CUU AGG CAC CAU tt and second siRNA sense: GUG UUC GCC AGU UAC UAC Att, Antisense: UGU AGU AAC UGG CGA ACA Ctt). 50 µM concentration were used for transfection with lipofectamine LTX. For real-time PCR, Total RNA was extracted with Tri reagent (Sigma). Reverse transcription was performed using SuperScript II following manufacturer protocol (Invitrogen). Real-time PCR was performed using taqman sets ordered from Applied Biosystems, Foster City, CA. As internal control, we used a VIC-labelled GAPDH mRNA probe (Applied Biosystems).

Western blot analysis was performed as previously described [24]. ABCE1 Rabbit polyclonal antibody, NB400-116 from Novus. Anti-HA, antibodies were obtained from Roche, respectively. Anti-β-actin anti-GAPDH were ordered from Cell Signaling Technology.

### Cell proliferation assay

Cellular proliferation was assessed using real-time label-free electric impedance system (ACEA) as described previously [9,25]. Briefly, HT-29 cells were seeded in a six well plate and transfected with 50 nM siRNA overnight. The cells were then re-seeded into 96-well electronic microtiter plate (E-plate) to ~60% confluence. The XCelligence real time cell analysis (RTCA) was performed to investigate cell proliferation. Growth was monitored for up to 25 hours with impedance assessed at 15 minutes intervals. Data were exported from the xCelligence system to GraphPad for visualization.

## Results

### Distribution of UU+UA dinucleotides in the human transcriptome

**Figure 1.**
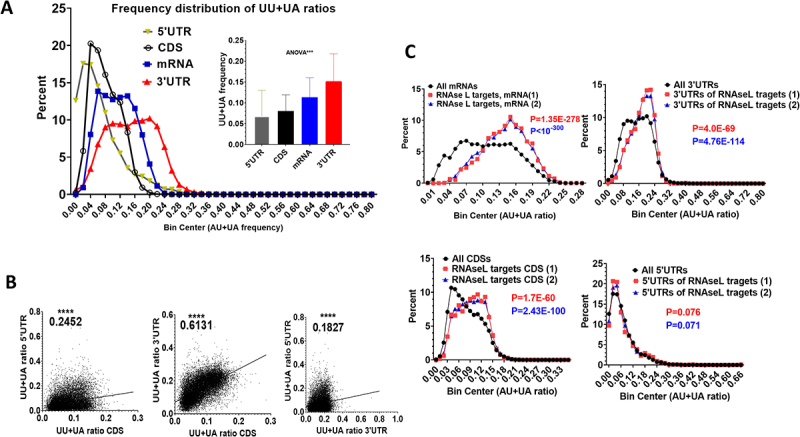
UU+UA dinucleotide ratios in the different regions of human mRNAs and targeting by RNase L (A) the sequences of human mRNAs and their different regions (complete sequence (mRNA), coding sequence (CDS), 5′ and 3′ untranslated regions (5′UTR and 3′UTR) were downloaded from ENSEMBL. The longest transcript for each gene was selected. The ratio of UU+UA dinucleotides was determined and a frequency distribution of UU+UA ratios was plotted for each region as labelled using GraphPad prism. Embedded are the means and SD of the UU+UA ratio for each region. (B) Linear regression of spearman correlations between the UU+UA ratios of different mRNA regions of human genes. (C) Two lists of RNase L targets in HELA cells lysates were determined based on read losses in RNA-seq after two methods of RNase L activation: (1) direct treatment of lysates with synthetic 2–5A or (2) treatment with a combination of purified recombinant RNase L and 2–5A [10]. The frequency distribution of the UU+UA ratio of the RNase L mRNA targets determined by the two methods (1 or 2) were compared with that of all mRNAs (upper left panel). That same analysis was repeated with the coding sequence (CDS) and the 3′ and 5′ untranslated regions (3′UTR and 5′UTR) as indicated. Unpaired parametric two tailed t-tests were used to compare the frequency in all genes to that in the genes that are RNase L targets.

UU+UA dinucleotides are the major targets of RNase L within the sequences of viral and cellular RNAs [26,27]. The reduction of UU+UA ratios in the coding domain sequence (CDS) of mRNAs is sufficient to increase the stabilization of mRNAs and enhance their expression to proteins [11]. Therefore, we explored the frequency distribution of the UU+UA ratios in the different regions of human mRNAs (Figure 1(A) and supplemental table 1). A frequency distribution plot of the CDS shows that few coding regions have UU+UA ratios of less than 0.02 or more than 0.12. Most abundant are the CDSs with UU+UA ratios ranging from 0.04 to 0.08. The complete mRNAs have higher UU+UA ratios compared to CDSs ranging from 0.07 to 0.2. The 3′UTRs have the highest UU+UA ratios ranging mainly between 0.08 and 0.24. The 5′UTRs are lowest in UU+UA ratios, in fact, around 2000 human 5′UTRs have no UU+UAs at all and very few have ratios of more than 0.08 (Figure 1(A)). The spearman correlations of the ratios between the regions were determined and were all positive and significant (Figure 1(B)). The correlations were weak, 0.18, between the 3′ and 5′UTRs and 0.25 between the CDS and the 5′ UTRs. The correlation between the CDS and 3′UTR was strong, 0.613 (Figure 1(B)). The correlations between all the regions are positive and significant; meaning that if one region is rich in UU+UA, the rest of the transcript is likely rich as well. Biologically, it can be interpreted that the entire combined sequence of a specific transcript determines whether it is an RNase L (UU+UA) target or not.

### The UU+UA ratio of the entire mRNA sequence is the superior predictor for RNase L targeting

Rath et al., induced the activity of RNase L in HELA cell lysates using two methods: (i) direct treatment of the lysate with synthetic 2–5A or (ii) treatment of the lysate with a combination of purified recombinant RNase L and 2–5A. RNA-seq procedures determined that UU+UA rich transcripts had higher RNase L-dependent read losses and identified putative mRNA-targets based on reduced levels due to RNase L activity [10]. We validated our assembled UU+UA ratio libraries against the putative RNase L targets from the mentioned study. A threshold of 0.5-fold reduction after RNase L activation was set to select RNase L targets for the two RNase L activation methods. The frequency distributions of the UU+UA ratios at a transcriptomic scale in mRNAs, CDSs, and 3′UTRs were compared to distributions in RNase L targets from Rath et al. As expected, the UU+UA ratios of RNase L targets shifted to a higher distribution than in all transcripts (mRNA) (Figure 1(C)). The CDS alone is also sufficient for a shift to a higher UU+UA frequency within the RNase L targets, which is in agreement with our previous findings [11]. The 3′UTRs of RNase L targets are richer in UU+UA dinucleotides compared to all 3′UTRs. The UU+UA ratio of the 5′UTRs had no statistically significant effect (Figure 1(C)). The most significant shift to a higher UU+UA frequency was observed with the whole mRNA. These findings indicate that the absolute number of the UU+UAs in the whole transcript is the best predictor for the level of RNase L targeting, and the whole mRNA sequence is best suited for further investigations.

### Reduced RNase L mRNA abundance in cancer does not associate with higher levels of UU+UA rich transcripts

**Figure 2.**
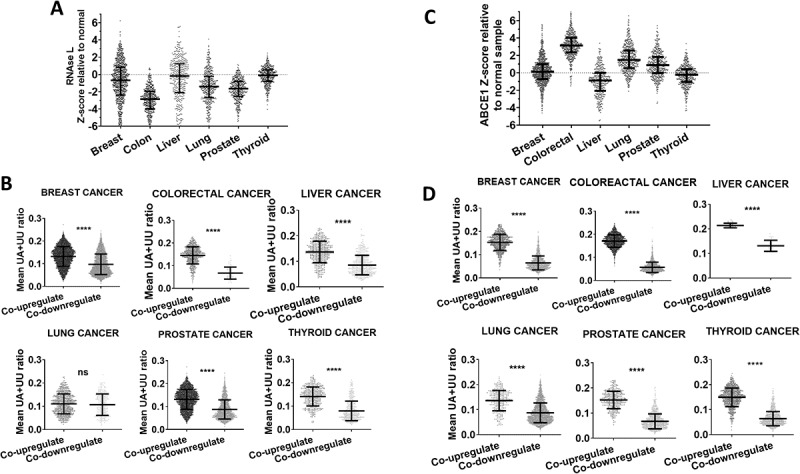
RNase L and ABCE1 mRNA levels in different cancers and the association with UU+UA ratios of co-expressed transcripts. (A) cBioportal was used to download RNase L mRNA levels (Z-values) relative to normal in the indicated cancer types. (B) mRNAs that co-overexpress and co-underexpress with RNase L were selected as described in methods. The UU+UA ratios of genes that are co-upregulated and those that are co-downregulated with RNase L were determined and plotted for comparison. Mann Whitney t-test. ****p < 0.0001. (C and D), same analysis as A and B but with ABCE1 levels. Mann Whitney t-test. ****p < 0.0001.

RNase L is generally considered a tumour suppressor that is deficient in cancers, especially prostate cancer [5,6]. For this analysis, we selected six cancer types of large PanCancer RNA-seq datasets from TCGA, namely Breast invasive carcinoma, colorectal adenocarcinoma, Liver hepatocellular carcinoma, Lung adenocarcinoma, Prostate adenocarcinoma and Thyroid carcinoma. The data indicates that, compared to normal samples, RNase L mRNA levels are deficient in the tested cancers, specifically in colorectal and prostate cancers (Figure 2(A)), and to a lesser extent in lung and breast cancers. A slight deficiency was observed in liver and thyroid cancers (Figure 2(A)). Because RNase L cleaves UU+UA sites, low RNase L levels in cancer should be expected to associate with an increase in mRNAs that have high UU+UA ratios. To test this hypothesis, we used cBioportal to select mRNAs that co-upregulate with RNase L by setting an upregulation threshold of 1.5 standard deviations (SD) above the mean of RNase L in cancer (RNase L mRNA >1.5, Q < 0.05.). In contrast, we also selected mRNAs that co-downregulate with RNase L mRNA levels (RNase L mRNA < −1.5, Q < 0.05). The UU+UA ratios of genes that are co-upregulated and those that are co-downregulated with RNase L were determined and plotted for comparison (Figure 2(B)). In almost all cancers analysed, the UU+UA frequency was higher in genes that were co-upregulated with RNase L. This is not in agreement with the biological function of RNase L that is expected to cleave and reduce the abundance of UU+UA rich transcripts. This may indicate that the RNase L activity is rather compromised in contrast to its mere expression levels. Therefore, we investigated the role of the RNase L activity inhibitor, ABCE1.

### Elevated oncogenic levels of the RNase L inhibitor ABCE1 associate with the overexpression of UU+UA enriched transcripts

A similar analysis to the one performed on RNase L above was repeated with its inhibitor ABCE1. First, we investigated the expression levels of ABCE1 across seven cancer types relative to normal samples. In contrast to RNase L, ABCE1 tends to be upregulated especially in colorectal cancer (Unlogged Median Z value ~9 SDs above normal) and more than 90% of patients have a higher expression than normal (Figure 2(C)). In lung cancer ABCE1 levels were ~3 SDs above normal, in breast cancer only slightly above normal, while in liver and thyroid it tends to be slightly under-expressed. We selected genes that co-upregulate or co-downregulate with ABCE1, using the same criteria as for RNase L above. The genes that co-upregulate with ABCE1 had a significant higher mean of UU+UA ratios than those that co-downregulate in all tested cancer types (Figure 2(D)). Similar, observations were made when the threshold of over or under expression was set to (±2 SD from the mean (data not shown). This outcome aligns with the biological activity of ABCE1. The combined findings of RNase L and ABCE1 suggests that RNase L activity in cancer is not primarily determined by the abundance of its mRNA, but rather the levels of its inhibitor, ABCE1. Based on this observation further analysis of the effect of RNase L/ABCE1 axis in cancer was based on the levels of ABCE1.

### Shifts in UU+UA ratios of transcripts based on their correlation with ABCE1 in cancer

**Figure 3.**
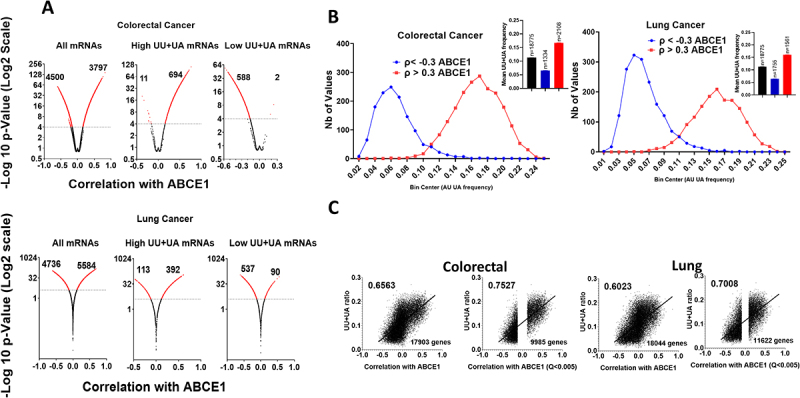
Shifts in UU+UA frequencies of transcripts based on their correlation with ABCE1 in colorectal and lung cancer (A) the spearman’s correlation (ρ) between ABCE1 mRNA and other mRNAs in colorectal and lung cancer was obtained from cBioportal along with the significance of this correlation (*p* value). The correlations were plotted against the -Log10 (*p* value) for all correlating mRNAs in volcano plots setting a threshold of *p* value of 0.0001 for significance (dotted line) (i.e. –Log10 = 4), the numbers of mRNAs that significantly (in red) positively or negatively correlate with ABCE1 mRNA are displayed. Next, the list was ranked according to UU+UA ratio of the mRNAs. The 1000 mRNAs with the highest UU+UA ratios were plotted in a volcano plot (-Log10 (*p* value) vs ρ value). The same was performed with 1000 mRNAs that have the lowest UU+UA ratio. Colorectal cancer: upper panel, lung cancer: lower panel. (B) Genes that have a positive correlation with ABCE1in colorectal cancer (ρ > 0.3) and those that have a negative correlation ρ < −0.3 were selected. The frequency distribution of the UU+UA ratio of the two set of genes was plotted using graph pad along with that of the whole transcriptome for comparison. Embedded are the means of the UU+UA ratios. The same analysis was performed for lung cancer as displayed. (C) the spearman’s correlation of human mRNAs with ABCE1 in colorectal and lung cancers was obtained from cBioportal and plotted against their UU+UA ratio. This was followed by linear regression and the determination of the correlation between the correlation of mRNAs with ABCE1 and their ratio of UU+UA. The same was repeated by selecting mRNAs with high statistical significance of their spearman’s correlation of their level with that of ABCE1 (Q < 0.005) (9985 in colorectal cancer and the 11,622 in lung cancer).

Since ABCE1 expression was highest in colorectal and lung cancers, we focused further studies on those two cancer types. The Spearman’s correlations (ρ) between ABCE1 mRNA and other mRNAs were uploaded from cBioportal TCGA Pan-cancer colorectal and lung studies, along with their statistical significance (P-value). The correlations were plotted against the -Log10 (*p* value) for all mRNAs in volcano plots, setting a threshold of *p* value of 0.0001 for significance (dotted line) (i.e. –Log10 = 4). In colorectal cancer, 3797 mRNAs correlated positively with ABCE1 and 4500 correlated negatively (Figure 3(A), upper panel, left). Subsequently, the mRNAs were ranked according to their UU+UA ratios (Supplementary table 1). The top 1000 genes with the highest UU+UA ratio and 1000 genes with the lowest ratio were subjected to the volcano plot analysis mentioned above. Of the 1000 genes with highest UU+UA ratio, 694 correlated positively with ABCE1 while only 11 correlated negatively, and the remaining mRNAs did not show significant correlation (Figure 3(A), upper panel, centre). Among the 1000 genes with the lowest UU+UA frequency, 588 mRNAs correlated negatively with ABCE1, while only two correlated positively, and the remaining mRNAs did not show significant correlation (Figure 3(A), upper panel, right). Similar observations were made in lung cancer (Figure 3(A) lower panel). These results represent a dramatic shift in UU+UA ratio based only on the correlation with ABCE1 in cancer.

In an alternative analytical setting, we selected genes that have a positive correlation with ABCE1 in colorectal cancer (ρ > 0.3) (2108 genes) and those that have a negative correlation ρ < −0.3 (1334 genes). Correlations starting at higher than 0.3 or lower than −0.3 are considered relevant in medical research [28]. We plotted a frequency distribution of the UU+UA ratio of the two sets of mRNAs. The results were striking: compared to each other and to the whole transcriptome, the genes that positively correlate with ABCE1 have a dramatic shift in distribution towards a high ratio of UU+UA from 0.12 to 0.24, and those that negatively correlate with ABCE1 have a dramatic shift towards a low ratio from 0.02 to 0.1 (Figure 3(B), left panel). The results align with the biological function of ABCE1 and suggest that the ABCE1/RNase L axis influence the relative levels of hundreds of genes. A similar analysis with similar results was observed in lung cancer (Figure 3(B), right panel).

**Figure 4.**
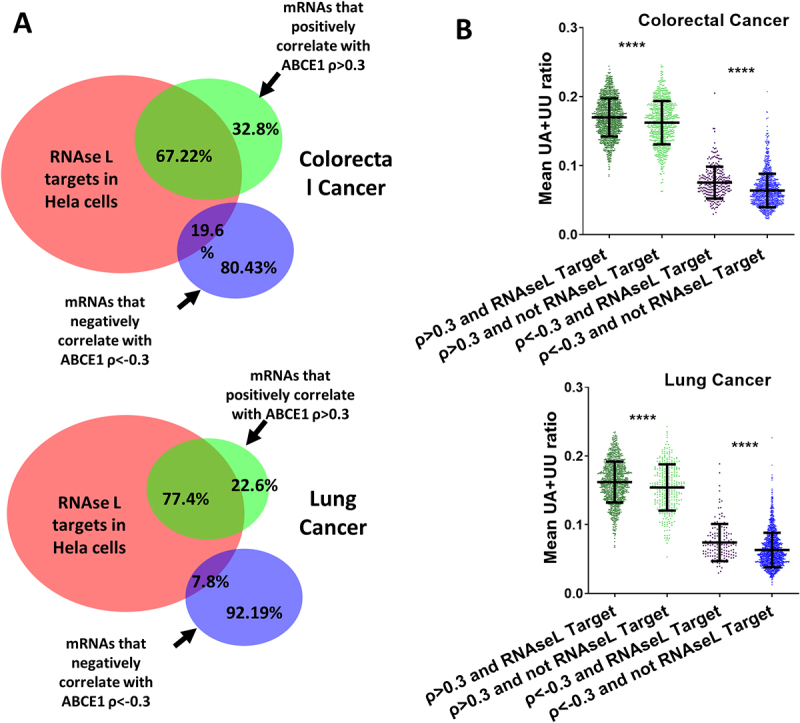
Intersection between RNase L targets identified in HELA cells and mRNAs that correlate with ABCE1 in cancer. The 5896 mRNAs that were identified as RNase L targets in HELA cells were intersected with mRNAs that positively or negatively correlate with ABCE1 ρ > 0.3 or ρ < −0.3 in colorectal and lung cancers. (A) Proportional venn diagrams illustrating the percentages of mRNAs that are common between RNase L targets in HELA cells and mRNAs that either positively or negative correlate with ABCE1 in colorectal (upper panel) or lung cancers (lower panel). (B) The UU+UA ratios of the different mRNA groups identified in a are displayed in colorectal cancer (upper panel), and lung cancer (lower panel). Mann Whitney t-test, ****p < 0.0001.

Afterwards, we determined the correlation between the UU+UA ratios in all mRNAs and their correlation with ABCE1 in colorectal and lung cancers. It turned out to be high, 0.66, and 0.6 respectively (Figure 3(C), left panel). Next, mRNAs with statistical significance of the spearman’s correlation of their level with that of ABCE1 (Q < 0.005) were selected (9985 in colorectal cancer and 11,622 in lung cancer). The correlations of the UU+UA frequency of those mRNAs with their correlation with ABCE1 mRNA levels were even higher, 0.75 and 0.7 respectively (Figure 4(C), right panel). These observations suggest that the correlative association of ABCE1 on mRNA transcripts is directly proportional to the UU+UA ratio over a wide spectrum of human protein coding mRNAs.

### Genes that positively correlate with ABCE1 in cancer are likely RNase L targets

RNase L targets are expected to positively correlate with ABCE1 in colorectal and lung cancers. To test this hypothesis, we used putative mRNA targets that were identified by Method 2 in HELA cells from Rath et. al. mentioned above [10]. A threshold of at least 0.5-fold reduction in level after RNase L activation was set to select putative RNase L targets resulting in a list 5896 mRNAs (Figure 4(A)). This list was crossed with mRNAs that positively correlate with ABCE1 in TCGA colorectal PanCancer (ρ > 0.3, 2108 mRNAs, generated in cBioportal) (Figure 4A, upper panel). Of the 2108 mRNAs, 1417 (67%) turned out to also be RNase L targets, which is a clear majority. In contrast, there were 1334 colorectal cancer mRNAs that negatively correlated with ABCE1 (ρ < −0.3) and only 261 were common with RNase L targets (19%), which is small fraction (Figure 4(A) upper panel). The same type of analysis was repeated in lung cancer with more striking results. For positively correlating mRNAs, 1209 out of 1562 were common with RNase L targets (77%), and only 137 out of 1755 were common in negatively correlating genes (7.8%) (Figure 4(A) lower panel). This clear reversal of abundance suggests that ABCE1 is a major regulator of RNase L activity in cancer. So far, the results were generated independently of the UU+UA frequency of the mRNAs and can be considered a cross validation of two fully independent sets of data. Finally, we plotted the UU+UA ratios of mRNAs that positively and negatively correlate with ABCE1 in the two cancer types. In addition, each correlating set was divided into two groups of RNase L targets and non-targets, resulting in four sets of mRNAs: (i) correlation with ABCE1 ρ > 0.3 and RNase L Target, (ii) ρ > 0.3 and RNase L Non-Target, (iii) ρ < −0.3 and RNase L Target, and (iv) ρ < −0.3 and RNase L Non-Target (Figure 4(B)). As expected, the UU+UA ratios were higher in mRNAs that positively correlate with ABCE1 compared with those that negatively correlate. However, and interestingly, even within each group, the targets of RNase L had a slight but significantly higher mean of UU+UA ratios (Figure 4(B)).

### Analysis of mRNAs that have a high correlation with ABCE1 and are rich in UU+UA

**Figure 5.**
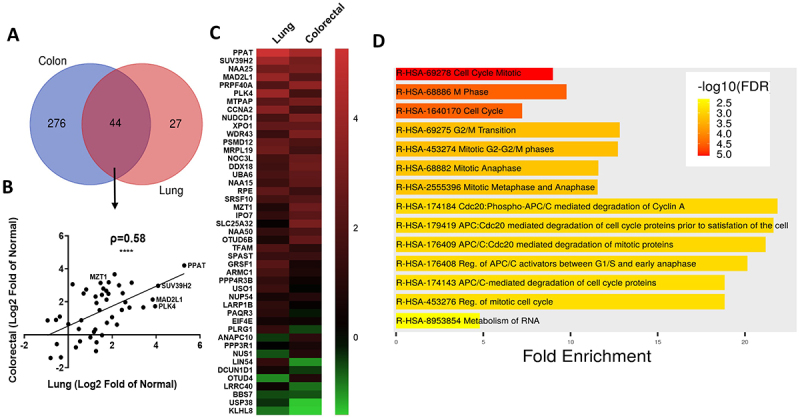
Analysis of mRNAs that correlate with ABCE1 and are high in UU+UA ratio. (A) Putative RNase L/ABCE1 targets were selected according to the following criteria: correlation with ABCE1 > 0.5 and UU+UA ratio > 0.16 resulting in 370 mRNAs in colorectal cancer and 71 in lung cancer. Venn diagram shows that 44 of the selected mRNA are common between colorectal and lung cancers. (B) plot of the correlation of the Log2 Fold change of normal between colorectal and lung cancers of the 44 common mRNAs. The correlation between values in lung and colorectal and the significance is displayed. (C) heat map displaying the expression levels of the 44 common genes in lung and colorectal cancers. (D) ShinyGO portal was used for gene enrichments analysis in the reactome category of the 44 common genes.

**Figure 6.**
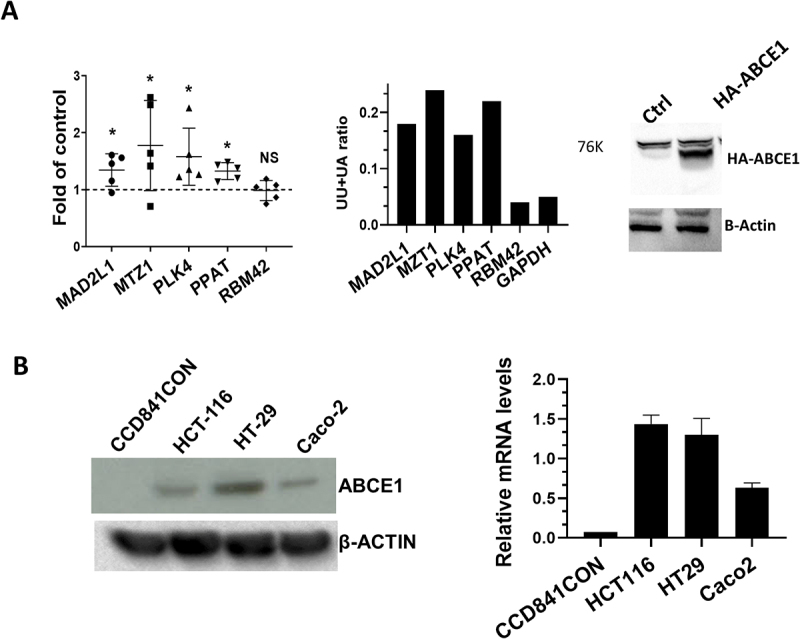
Effect of ABCE1 level modulation on UU+UA-rich mRNAs levels and on cell proliferation. (A) Left panel, HEK293 cells were transfected with HA-tagged ABCE1 or empty pcDNA 3.1 vector for 24 hours. Taqman qRT-PCR of the designated mRNAs normalized against GAPDH was performed, data shown in Fold of empty vector control of the five independent experiments *p < 0.05. NS, not significant. Middle panel, the ratio of the UU+UA of the investigated mRNA is displayed. Right panel, western blot of HEK293 lysates transfected with HA tagged ABCE1 or empty vector. (B) Left panel, western blot analysis of ABCE1 levels in normal colon cell line CCD841CON and CRC cell lines HCT116, HT29 and Caco-2. Right panel qPCR of ABCE1 in the same cell lines.

The following criteria were used to select mRNAs that are likely to be putative RNase L/ABCE1 targets in lung and colorectal cancers: we considered UU+UA ratio >0.16 as high based on the frequency distribution of UU+UA ratios (Figure 1(A)) and a robust positive correlation with ABCE1 >0.5 [28]. This method resulted in the selection of 320 genes in colorectal cancer and 71 genes in lung cancer (Supplemental table 2). The gene ontology of the selected mRNAs was analysed in Shiny Go 0.82 (http://bioinformatics.sdstate.edu/go/). Most interesting enrichments were observed in reactome pathways category: the strongest and most significant enrichments were related to cell cycle control, both in lung and colorectal cancers (Supplemental Figure 2). A Venn diagram shows that 44 of the mRNAs are common between the two cancer types (62% of mRNAs in lung cancer) (Figure 5(A)). Almost all of the common mRNAs are upregulated in both cancers and their log2FC relative to normal strongly correlate (ρ = 0.58), suggesting common dysregulation related to ABCE1 overexpression across cancer types (Figure 5(B, C)). The 44 common genes were also subjected to reactome pathways analysis. Most enriched categories relate to cell cycle, Mitotic control and include genes such as NUP54, PLK4, MAD2L1, PSMD12, XPO1, USO1, ANAPC10 and SPAST (Figure 5(D)). ABCE1 levels correlated positively with the levels of high UU+UA transcripts, not only in colorectal and lung cancer but in all of the six cancers tested in this study, even when ABCE1 is not over expressed (data not shown). These observations suggest that the effect may be universal of at least can be observed in a wide range of cancers and cell types. Therefore, we tested the effect of ABCE1 overexpression in the model of HEK293 cells due to the ease of transfection. HA-tagged ABCE1 was overexpressed in HEK293 cells and the levels of mRNAs that are rich in UU+UA and have a high correlation with ABCE1 in cancer were assessed by qPCR. The UU+UA rich mRNAs MAD2L1, MTZ1, PLK4, PPAT were upregulated whereas RBM42, which is low in UU+UA, was not significantly affected by ABCE1 overexpression (Figure 6(A)). In agreement with clinical observations, the levels of ABCE1 protein and mRNAs were elevated in the CRC cell lines HCT116, Caco-2 and HT29 compared to the normal colon cell-line CCD841CON (Figure 6(B)).

### Survival analysis of colorectal and lung cancer patients with high ABCE1

**Figure 7.**
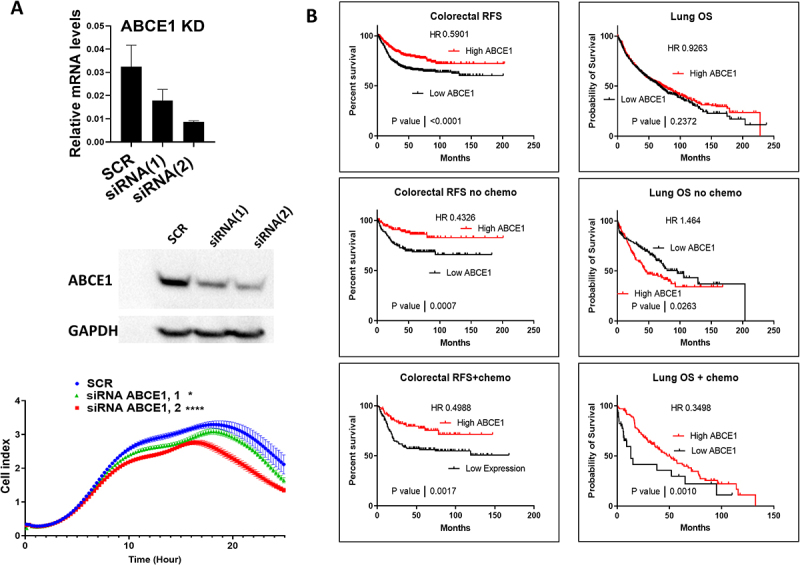
Effect of ABCE1 levels on cell proliferation and association with patient survival in colorectal and lung cancers. (A) The effect of ABCE1 knock-down on HT29 CRC cell proliferation. Two independent siRnas were used to knock-down ABCE1 in HT29 cells. Upper panel, the effect of the knock-down on ABCE1 mRNAs levels by qPCR. Middle panel, the effect of the knock-down on ABCE1 protein levels by western blot. Lower panel, continuous monitoring of cell proliferation of HT29 colon cancer cells that were transfected with control siRNA (SCR) or siRnas that targets ABCE1 expression. The experiments consist of three biological replicates and is representative of two independent experiments with comparable results. Mann-Whitney t -test ****p < 0.0001, *p < 0.05. (B) Survival curves for colorectal and lung cancer patients were generated using the KM plotter http://kmplot.com. Patients were split based on best cutoff expression levels of ABCE1 (JetSet probe). Left: colorectal cancer. Upper left panel: the relapse free survival (RFS) of all colorectal patients. Middle left panel RFS of colorectal patients that did not receive chemotherapy. Lower left panel RFS of colorectal cancer patients that received chemotherapy. Upper right panel: overall survival (OS) of all lung cancer patients. Middle right panel, OS of lung cancer patients that did not receive chemotherapy. Lower right panel: OS of lung cancer patients that received chemotherapy. HR hazardous ratio.

The Konckdown of ABCE1 levels in HT29 cells by two independent interfering dsRNAs resulted in reduced cell growth, similar to observations in colon and lung cancer cells (Figure 7(A)) [29–31]. ABCE1 is highly overexpressed in colorectal cancer with a mean expression ~9 SDs more than normal. This extreme overexpression is much higher than in all other investigated cancers, including lung cancer which comes second (median ~3 fold of normal) (Figure 2(C)). Previous reports indicate that ABCE1 upregulation drives cell division and cancer progression in lung cancer [15,16]. Therefore, we investigated the effect of this overexpression on patient survival using the KM plotter portal [32]. Unexpectedly, in colorectal cancer, high ABCE1 was associated with a significant favourable outcome, in terms of Relapse Free Survival (Figure 7(B), left panel up). Next, we separated colorectal cancer patients according to chemotherapy treatment, still high ABCE1 levels led to favourable patient outcomes in all cases (Figure 7(B), left panel middle and down). These surprising results may be related to an interesting previous report: Shichijo et al. found out that ABCE1-derived peptides are presented as HLA-A2-restricted tumour antigens in colorectal cancer calls and were recognized by cytotoxic T-cells (CTL) [33]. The extreme upregulation of ABCE1 might be triggering an immune response that leads to an improved patient outcome. However, this hypothesis remains speculative, and further mechanistic and functional studies will be required to validate this association and establish a causal relationship.

Next, the effect of ABCE1 expression on overall survival of lung cancer patients was also investigated (Figure 7(B), right panel). Among all patients, no statistically significant association of high ABCE1 with overall survival could be observed. ABCE1 drives cell division, and rapidly dividing lung cancer cells should be more susceptible to chemotherapy treatment than slower dividing cells [15,16]. Therefore, we investigated the effect of ABCE1 expression on overall survival of lung cancer treated with chemotherapy and those with no chemotherapy treatment. Interestingly, overexpression of ABCE1 was associated with favourable patient outcome in chemotherapy treated patients but was unfavourable in patients with no chemotherapy treatment (Figure 7(B), right panel, middle and down). The results align with the ability of ABCE1 to drive cancer progression and cell division. Its overexpression in cancer is unfavourable since its drives cell division and cancer progression, but for the same reason it becomes favourable when patients are treated with chemotherapy. In conclusion, we propose that ABCE1 is in fact a tumour driver, however, also a possible cancer associated antigen in colorectal cancer. This latter property overrides the negative effects of its cell division promoting effect.

## Discussion

In this study, we report that the upregulation of a single factor, namely the RNase L inhibitor ABCE1, is associated with observable effects on the oncogenic transcriptome: it apparently leads to an extensive ‘transcriptomic shift’ towards higher levels of hundreds of mRNAs that are rich in UU+UA dinucleotides. This shift is likely to have significant biological consequences on cancer cells, including modulation of cell cycle progression, since several of the most upregulated UU+UA rich mRNAs relate to cell cycle and mitotic phase. Our observations align with previous findings that associate the overexpression of ABCE1 to proliferation and invasiveness of cancer cells [15,16,29].

The mRNA levels of RNase L and ABCE1 were investigated in six TCGA cancer types, namely, breast invasive carcinoma, colorectal adenocarcinoma, liver hepatocellular carcinoma, lung adenocarcinoma, prostate adenocarcinoma and thyroid carcinoma. In agreement with previous reports, we observed a reduction of RNase L mRNA levels in cancer compared to normal [12,13]. This RNase L downregulation, however, did not associate with an upregulation of its putative targets: the UU+UA rich transcripts. In contrast, the upregulation of ABCE1 correlated with an upregulation of UU+UA rich transcripts on a wide scale and in all cancers that were investigated, in agreement with its biological function. Consequently, ABCE1 level must be the main controlling factor that determines the extent of the activity of the RNase L pathway in cancer. This finding aligns with a previous report stating that high levels of RNase L in NCI-H157 lung cancer cells remain inactive due to ABCE1 inhibition [34]. Most of the mRNAs that positively correlate with ABCE1 in colorectal and lung cancers turned out to be also targets of RNase L that were identified in HELA cells [10]. The agreement between these fully independent data sets strengthens the validity of the cancer findings and implies that the biological effect of ABCE1 in cancer is exerted by the inhibition of RNase L.

The scale of the observed putative effect of ABCE1 oncogenic upregulation appears to be wide. The difference in the distribution of UU+UA ratio was high between mRNAs that positively or negatively correlate with the mRNA of ABCE1. In fact, the correlation between the correlation of cancer mRNAs with ABCE1, and their own UU+UA ratio was positive and high (up to 0.75) over a wide range of the transcriptome.

This study focused on the analysis of the cancer types where ABCE1 was most upregulated, i.e. colorectal and lung, however, comparable observations were made in other cancer types even when ABCE1 was weakly upregulated or even downregulated. For instance, the mean ABCE1 level in breast cancer is only slightly above normal and even below normal in thyroid cancer. Still, in both cancers, patients that have higher levels of ABCE1 have also significantly higher levels of UU+UA rich transcripts.

Amongst the cancers tested, ABCE1 was especially upregulated in colorectal adenocarcinomas, median approximately ninefold higher than normal and in many patients more than 20 and 100-fold higher than normal. However, and surprisingly at first, this upregulation was associated with a favourable relapse free survival in colorectal adenocarcinoma according to Kaplan Meier survival analysis. Previous reports showed that ABCE1-derived HLA-A2-restricted cancer antigens have been identified in colorectal cancer patients. ABCE1 peptides triggered the generation of HLA-A2-restricted and colorectal cancer-reactive CTLs from the PBMCs of colorectal cancer patients, but not from healthy donors [33]. It is likely that the oncogenic/mitogenic effects of ABCE1 reported here and elsewhere in cancer exists in colorectal cancer, i.e. ABCE1 upregulation leads to UU+UA dependent shift on the cancer transcriptome resulting in enhanced cell division and cancer progression. This detrimental abnormality, however, may be detected by the immune system turning ABCE1 overexpression into a positive prognostic factor, similar to colorectal cancers with microsatellite instability [35]. In lung cancer, and perhaps due to its lesser level of overexpression, ABCE1 presents a stealthier target for the immune system. Nonetheless, the analysis of ABCE1 effect on overall survival in lung cancer was not straightforward. The overexpression of ABCE1 in all lung cancer patients did not have a statistically significant effect on overall survival. However, in patients not treated with chemotherapy, ABCE1 was a negative prognostic factor, in agreement with its effect on promoting cell division. However, in lung cancer patients that were treated with chemotherapy, ABCE1 became a positive prognostic factor. This is again in agreement with ABCE1’s ability to drive cell division since chemotherapy targets rapidly dividing cells.

The steady state levels of cellular mRNAs are the result of complex regulatory networks including the regulation of transcription, processing, transport and stability [36]. This process depends on default or modifiable elements like regulatory sequence elements, signalling cascades, structures in DNA and RNA, chromatin remodelling and epigenetics [37–40]. As a result, each gene is likely to have its unique fine-tuned regulation that is dependent on these combined factors. Amidst this overwhelming background, we propose, that the ABCE1/RNase L axis alone may trigger a measurable global oncogenic ‘transcriptomic shift’ with likely consequences on cancer severity and progression.

## Supplementary Material

Supplemental sheet1 UUUA lists.xlsx

EnrichementsColon and Lung cancers suppl figure 1.tif

Supplemental Sheet 2.xlsx

## Data Availability

All data supporting the findings of this study are available within the article and its supplementary materials or corresponding author upon reasonable request.
