# Supplementary figures and images for: Profound alterations of cancer transcriptomes by the RNase L inhibitor ABCE1 through the modulation of UU/UA-dinucleotide rich transcript abundance
Source: RNA Biol. 2026 Feb 9;23(1):1–14. doi: 10.1080/15476286.2026.2629475 (PMC12915867; doi:10.1080/15476286.2026.2629475)

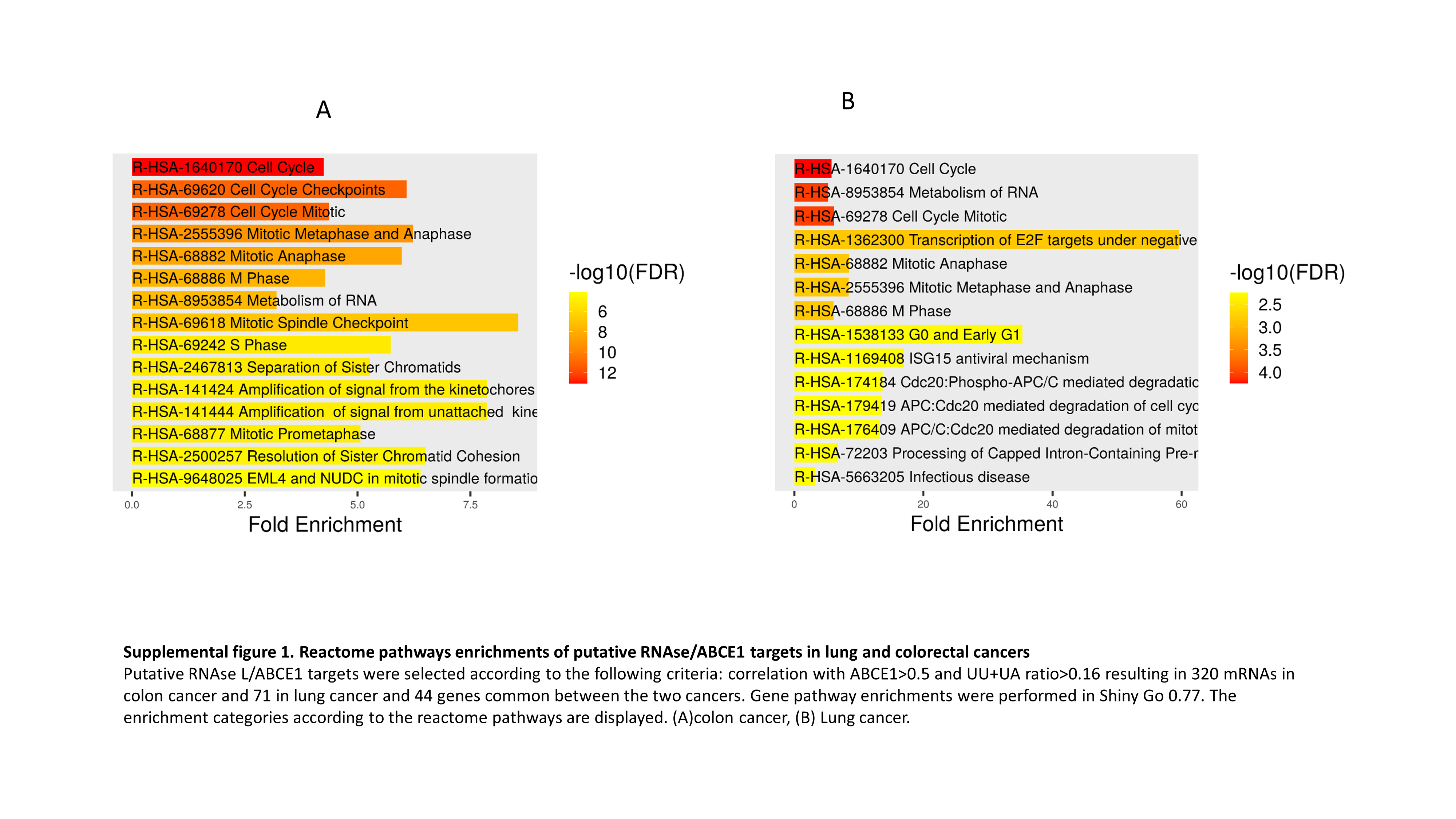

Supplement: EnrichementsColon and Lung cancers suppl figure 1.tif [file KRNB_A_2629475_SM3983.tif]
